# Pericardial-Peritoneal Window as an Alternative Treatment for Large and Recurrent Pericardial Effusion Post-Pericardiotomy

**DOI:** 10.21470/1678-9741-2020-0355

**Published:** 2021

**Authors:** Luis Roberto Palma Dallan, Luis Alberto Oliveira Dallan, Omar Vilca Mejía, Luis Augusto Palma Dallan, Luiz Augusto Ferreira Lisboa, Fabio B. Jatene

**Affiliations:** 1 Department of Cardiovascular Surgery, Instituto do Coração do Hospital das Clínicas da Faculdade de Medicina do Estado de São Paulo (InCor), São Paulo, São Paulo, Brazil.

**Keywords:** Pericardial Effusion, Pericardial Window Techniques, Cardiac Tamponade, Patient Discharge, Cardiac Surgical Procedures, Coronary Artery Bypass, Intraoperative Complications

## Abstract

**Introduction:**

The presence of mild to moderate pericardial effusion after cardiac surgery is common and oral medical therapy is usually able to treat it. Larger effusions are less frequent and surgical intervention is usually necessary. However, there are some rare cases of large effusions that are recurrent even after intervention and become challenging to treat.

**Methods:**

We describe the case of a patient submitted to coronary artery bypass grafting (CABG) without any intraoperative complications, who was regularly discharged from the hospital. She was referred to our emergency department twice after surgery with large pericardial effusion that was drained. Even after those two interventions and with adequate oral medication, the large effusion recurred.

**Results:**

During follow-up, the patient had her symptoms resolved, with no need for further hospital admission. Her echocardiograms after the last intervention showed no pericardial effusion. The present surgical technique demonstrated to be easy to perform, thus it should be considered as a treatment option for these rare cases of large and repetitive effusions, which do not respond to the traditional methods.

**Conclusions:**

In challenging cases of recurrent and large pericardial effusions, the pericardial-peritoneal window is an alternative surgical technique that brings clinical improvement and diminishes the risk of cardiac tamponade.

**Table:** 

Abbreviations, acronyms & symbols	
**CABG**	**= Coronary artery bypass grafting**
**CCS**	**= Canadian Cardiovascular Society**

## INTRODUCTION

We describe a case of a recurrent large pericardial effusion post-pericardiotomy, not responsive to usual drug therapy, in which we performed an alternative surgical method for its management.

## CASE DESCRIPTION

A 65-year-old female patient with symptoms of stable angina (Canadian Cardiovascular Society [CCS] class 2), without previous hospitalizations, underwent an elective coronary artery bypass grafting surgery (CABG) with cardiopulmonary bypass. Pericardial friction rub was not present on her cardiac auscultation before surgery. The following grafts were used: left internal thoracic artery for the anterior interventricular branch of the left coronary artery, saphenous vein graft for the left coronary marginal branch, and saphenous vein graft for the right coronary artery. The patient had a good initial clinical evolution, being discharged on the 8^th^ postoperative day.

However, after 20 days, symptoms of respiratory distress with progressive dyspnea appeared. Echocardiographic examination revealed significant pericardial effusion. The patient was submitted to subxiphoid pericardial drainage and pericardial biopsy, with removal of about 600 ml of serosanguineous fluid and installation of local drainage. Since the condition corresponded to an inflammatory response after surgery, we decided to remove the pericardial chest tube on the 3^rd^ day, when the daily drainage amount was less than 100 ml. The patient was discharged asymptomatic, receiving prednisolone (40 mg/day).

Pericardial biopsy and immunological study revealed a nonspecific inflammatory process, without biochemical alterations, suggesting chylous effusion, also with negative testing for mycobacteria, herpes and cytomegalovirus.

After three months, the patient presented symptoms of dyspnea for minor efforts, although she did not have other symptoms like fever or asthenia. The echo images showed significant pericardial effusion and she underwent new pericardial drainage.

The pericardial effusion remained serosanguineous. The postoperative chest tube evolved with significant drainage about 500 ml/day in the first week. We raised the hypothesis of thoracic duct injury that could justify such volume during this week, but not only the macroscopic aspect of the drainage was serosanguineous, but also laboratorial tests were negative for triglycerides.

The medical treatment consisted of corticosteroids, diuretics and hydrosaline restriction. After 7 days, the daily volume decreased probably due to a good response to clinical treatment and could be removed on the 10^th^ day and the patient was discharged home. Again, the pericardial liquid revealed a nonspecific inflammatory process.

However, after two more months, a third episode of pericardial effusion occurred, with the patient presenting symptoms of low cardiac output. At this point, we decided on an alternative surgical procedure creating a pericardial-peritoneal window (as described in the technique part).

## TECHNIQUE

**Fig. 1:**
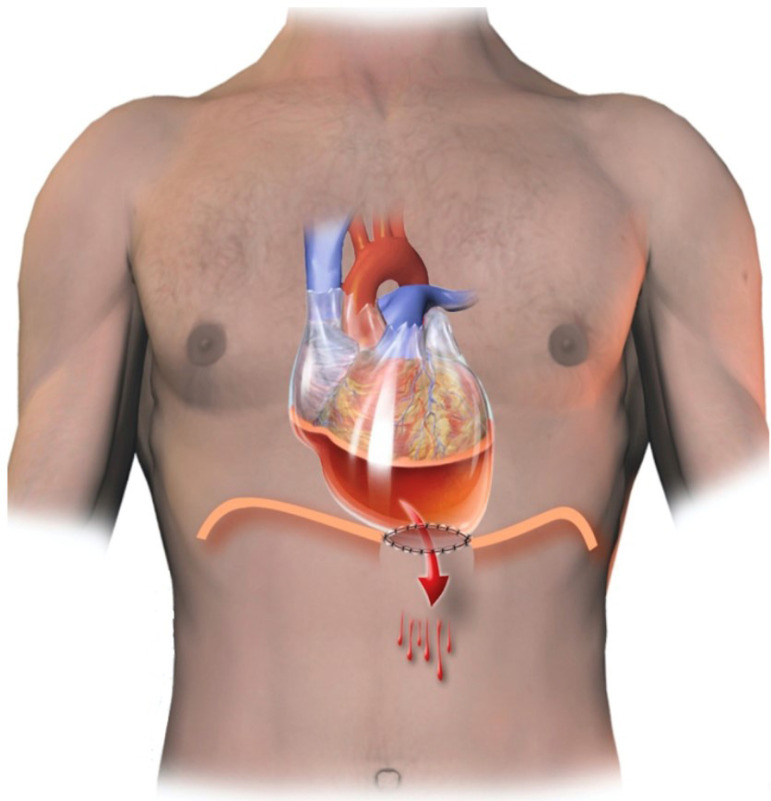
Schematic figure of the window created between the tissues of the pericardial and peritoneal cavities.

Under sedation, supplemented by local anesthesia, a subxiphoid incision of about 5 centimeters in length was made. The pericardial cavity was accessed, and 800 ml of serous fluid was aspirated. This was followed by a 4-cm incision in the peritoneum, in the region corresponding to the diaphragmatic opening of the pericardium. To prevent the closure of this orifice, the tissue borders of the pericardial-peritoneal window were sutured using continuous 4-0 catgut (Figure 1). No local drainage was performed.

## DISCUSSION

The presence of pericardial effusion and its treatment have been the object of many studies^[^^1^^,^^2^^]^. The presence of mild pericardial effusion after cardiac surgery often results from post-pericardiotomy inflammatory syndrome and presents adequate regression with clinical drug treatment.

However, in a small number of cases, large pericardial effusion can be recurrent, requiring multiple pericardial drainage procedures. Albeit surgical drainage is easy to perform, the repetition of this procedure brings discomfort to the patient and a definitive resolution is necessary.

These repetitive effusions are more frequently seen in patients with chylothorax due to disruption of the lymphatic vessels or thoracic duct, but in these cases, the macroscopic findings are milky effusion with high levels of triglycerides^[^^3^^]^.

It is important to mention that, even in the case of a chylous effusion caused by surgical trauma, spontaneous resolution occurs in up to 50% of the cases^[^^4^^]^. Nonsurgical treatment can sometimes be achieved with dietary measures with a low-fat diet associated with medium-chain triglycerides. In refractory cases, a parenteral diet is recommended.

Surgery would be indicated in case of clinical failure after two weeks. A pleural-peritoneal shunt as a surgical strategy has been previously suggested, similarly to our pericardial-peritoneal shunt described here^[^^4^^]^. Once we ruled out the hypothesis of chylous effusion after laboratory tests, the inflammatory cause was our main hypothesis. As mentioned, the patient has already been using corticosteroids, 40 mg/day since her second episode, therefore, pulse therapy was not considered by the cardiologists.

Although we consider surgery the trigger of the inflammatory cause, it is unlikely that, after a long period, surgical trauma would maintain such an intense response. Unfortunately, seeking the basis of this problem has been challenging and has brought frustration to the case so far. We are faced to find a necessary resolution for hospital readmissions.

For similar cases of large repetitive pleural effusion, some authors have reported the creation of a window between the pericardium and the pleural cavity, especially as a palliative solution when the pathogenicity is due to a malignant tumor^[^^5^^-^^7^^]^.

During the heart team discussion, we decided to create the pericardial-peritoneal window, taking into account that the abdominal cavity is more compliant to volume and has greater fluid absorption than the pleural cavity. This would reduce the possibility of a new episode of large pericardial effusion and its life-threatening consequences caused by low cardiac output.

We believe that, maintaining adequate oral medical treatment and removing the chances of a life-threatening event, the inflammatory cause would cease with time and the small amount of fluid would be absorbed in the peritoneal cavity.

After the last surgical approach, the patient had a good recovery, without cardiovascular symptoms even after a one-year follow-up. Successive control echocardiograms no longer detected pericardial effusion. In addition, she had no abdominal complains or abdominal ascites on physical examination.

## CONCLUSION

The case presented here shows that a pericardial-peritoneal window may be recommended as an alternative treatment for recurrent pericardial effusion post-pericardiotomy.

**Table:** 

Authors' roles & responsibilities	
LRPD	Substantial contributions to the conception or design of the work; or the acquisition, analysis or interpretation of data for the work; final approval of the version to be published
LAOD	Substantial contributions to the conception or design of the work; or the acquisition, analysis or interpretation of data for the work; drafting the work or revising it critically for important intellectual content; final approval of the version to be published
OVM	Drafting the work or revising it critically for important intellectual content; final approval of the version to be published
LAPD	Drafting the work or revising it critically for important intellectual content; final approval of the version to be published
LAFL	Drafting the work or revising it critically for important intellectual content; final approval of the version to be published
FBJ	Drafting the work or revising it critically for important intellectual content; final approval of the version to be published
